# Integrated single-cell and bulk RNA sequencing analysis identifies a cancer-associated fibroblast-related gene signature for predicting survival and therapy in gastric cancer

**DOI:** 10.1186/s12885-022-10332-w

**Published:** 2023-01-31

**Authors:** Zhiyang Zhou, Sixuan Guo, Shuhui Lai, Tao Wang, Yao Du, Junping Deng, Shun Zhang, Ge Gao, Jiangnan Zhang

**Affiliations:** 1 Department of General Surgery, First Affiliated Hospital of Nanchang University, Nanchang, Jiangxi Province China; 2 Nanchang University, Nanchang, Jiangxi Province China; 3 Department of Day Ward, First Affiliated Hospital of Nanchang University, Nanchang, Jiangxi Province China

**Keywords:** Cancer-associated fibroblasts, Gastric cancer, Single-cell RNA sequencing, Gene signature, Small molecular drug, Tumor microenvironment

## Abstract

As the dominant component of the tumor microenvironment, cancer-associated fibroblasts (CAFs), play a vital role in tumor progression. An increasing number of studies have confirmed that CAFs are involved in almost every aspect of tumors including tumorigenesis, metabolism, invasion, metastasis and drug resistance, and CAFs provide an attractive therapeutic target. This study aimed to explore the feature genes of CAFs for potential therapeutic targets and reliable prediction of prognosis in patients with gastric cancer (GC). Bioinformatic analysis was utilized to identify the feature genes of CAFs in GC by performing an integrated analysis of single-cell and transcriptome RNA sequencing using R software. Based on these feature genes, a CAF-related gene signature was constructed for prognostic prediction by LASSO. Simultaneously, survival analysis and nomogram were performed to validate the prognostic predictive value of this gene signature, and qRT–PCR and immunohistochemical staining verified the expression of the feature genes of CAFs. In addition, small molecular drugs for gene therapy of CAF-related gene signatures in GC patients were identified using the connectivity map (CMAP) database. A combination of nine CAF-related genes was constructed to characterize the prognosis of GC, and the prognostic potential and differential expression of the gene signature were initially validated. Additionally, three small molecular drugs were deduced to have anticancer properties on GC progression. By integrating single-cell and bulk RNA sequencing analyses, a novel gene signature of CAFs was constructed. The results provide a positive impact on future research and clinical studies involving CAFs for GC.

**Supplementary Information:**

The online version contains supplementary material available at 10.1186/s12885-022-10332-w.

## Background

The latest statistics for GLOBOCAN 2020 showed that gastric cancer (GC) was the fifth most frequently diagnosed cancer, and the incidence is especially high in Eastern Asian countries, and it is extremely harmful with mortality rate that ranks the fourth in cancer-related death after lung cancer, colorectal cancer, and liver cancer [1, 2]. Following the successful application of targeted therapy and immunotherapy in clinical practice, we present a novel strategy in advanced GC. However, the therapeutic efficacy did not achieve the desired improvement, which emphasizes the need to focus not only on the tumor cells themselves but also on the significance of the surrounding environment, the tumor microenvironment (TME), which comprises all nontumor cells and their noncellular components, such as the extracellular matrix (ECM) and soluble molecules [3]. The crosstalk between tumor cells and the TME directly influences tumor cell growth and cancer progression. Among the different nontumor cells, cancer-associated fibroblasts (CAFs) deserve special attention.

Activated fibroblasts, also defined as CAFs, have long been considered to coevolve with tumor cells as the dominant component of the tumor stroma [4]. CAFs secrete a variety of cytokines, growth factors and chemokines that form fertile soil for the growth of tumor cells; for example, CAFs secrete interleukin-6 (IL-6) or interleukin-11 (IL-11), resulting in tumor progression and the development of chemotherapeutic resistance [5–7]. In turn, tumor cells secrete numerous factors such as transforming growth factor-β (TGF-β), epidermal growth factor (EGF) and C-X-C motif chemokine ligand 12 (CXCL12), which can activate and educate CAFs [8]. Accumulating studies have confirmed that CAFs are involved in almost every aspect of tumors, including tumorigenesis, metabolism, invasion, metastasis and drug resistance, and CAFs provide an attractive therapeutic target [9–11].

Notably, researchers are presently unable to achieve breakthroughs in developing viable therapies for CAFs owing to the highly dynamic heterogeneity of CAFs. Indeed, CAFs have diverse potential cellular origins, including resident fibroblasts, mesenchymal stem cells, adipocytes, epithelial cells, mesothelial cells and endothelial cells, and form various subpopulations in different tumor types [8, 10, 12]. Additionally, CAF heterogeneity could possibly be the result of a common precursor in cells at various stages of differentiation that have adopted distinct states based on signaling cues both inside and outside the TME. Currently, α-smooth muscle actin (αSMA), fibroblast-specific protein 1 (FSP1), fibroblast activation protein (FAP), platelet-derived growth factor receptor-α (PDGFRα), PDGFRβ, discoidin domain-containing receptor 2 (DDR2), insulin-like growth factor-binding protein 7 (IGFBP7), caveolin‐1 (CAV1), CD90 (Thy1), tenascin‐C (TNC), periostin (POSTN), podoplanin (PDPN), decorin (DCN), desmin, vimentin and integrin β1 are considered activated CAF markers, and no single specific biomarker can categorize the whole CAF population or distinguish CAFs from all other cell types [8, 10, 13, 14]. As a result, identifying CAFs is extremely difficult and poses a huge challenge for targeted treatment of CAFs.

Additionally, the exploration of the prognostic value of CAFs is also an important reference for individualized treatment, and numerous studies have attempted to validate CAFs as potential pathological indicators of tumor prognosis. In this regard, αSMA serves as a hallmark of prognostic factors. Immunohistochemical (IHC) staining analysis of hepatocellular carcinoma (HCC) patients shows a significantly shorter disease-free survival rate in patients with tumors overexpressing α-SMA [15, 16], and the same negative correlation was shown in colorectal cancer (CRC) and breast cancer [17, 18]. Furthermore, the differential expression signatures of specific genes in CAFs can be used as prognostic tools. In CRC research, Alexandre et al. revealed that high expression levels of the 4-gene signature identify patients with poor prognosis in the CAF cluster [19]. Zou et al. also reported a 12-gene signature of CAFs and its high expression was significantly correlated with pathological and increased clinical events of tumor progression of HCC [20]. However, these CAF-related signatures do not overlap, which presents the same nonspecificity concern in the application of CAFs. Therefore, further clarification of the relationship between CAFs and prognosis and its value in predicting survival will also accelerate the transition from basic CAF research to clinical application. Therefore, the exploration of new biomarkers of CAFs will be of significance.

In this study, we aimed to identify the gene signature of CAFs in GC by performing an integrated analysis of single-cell RNA sequencing (scRNA-seq) and transcriptome RNA sequencing (RNA-seq) with data from The Cancer Genome Atlas (TCGA) and Gene Expression Omnibus (GEO). Based on CAF-related genes, we constructed a risk score for prognostic prediction by LASSO, and the analysis revealed that the risk score can be an independent prognostic factor. Then, we established a nomogram model to perform quantitative scores derived from the risk score and other clinicopathological features. In addition, we aimed to identify promising small molecular drugs for gene therapy of CAF-related gene signatures in GC patients.

## Materials and methods

### Data acquisition

We downloaded the expression matrix of 414 GC and 36 normal gastric samples, and 387 GC samples contained overall survival (OS) data. The clinical information included age, gender, pathologic stage, grade and fraction genome altered, which were procured from the UCSC Cancer Genomics Browser. As a validation set, the GSE62254 dataset including 300 GC samples was downloaded, simultaneously containing OS information generated via the GPL570 platform. In addition, we downloaded the single-cell transcriptome expression profiles of 158,641 cells in 40 samples (29 GC samples and 11 normal samples) from GSE183904 via the GEO database.

### Estimation of immune infiltration

The Microenvironment Cell Populations-counter (MCP-counter) package has been applied to study the cellular composition of the microenvironment [21], which uses the gene expression matrix to produce the scores of immunocytes and stromal cells [22]. Therefore, the mRNA data were translated into nontumor cell infiltration levels within the TME using the MCP-counter package of R software.

### Processing of single-cell RNA-seq data

We generated a “Seurat” object based on the transcriptome sequencing data of 158,641 cells using the “Seurat” package [23]. The top 2000 genes with highly variable features accounting for cell-to-cell differences were identified by variance analysis and subjected to data scaling and centering. These variable genes were further used for principal component analysis (PCA) with linear dimensionality reduction. The top 35 principal components (PCs) were applied for graph-based clustering (res = 0.4) to identify distinct groups of cells. The cell clusters were visualized based on the “UMAP” method of dimensionality reduction. Clusters were annotated through the “SingleR” package based on the reference gene list of 713 samples from the “HumanPrimaryCellAtlasData” function [24].

### Risk assessment model construction and evaluation

In the creation of innovative clinical prediction models, the least absolute shrinkage selection operator (LASSO) regression model is typically utilized [25]. Based on the gene signature generated by LASSO, we calculated the risk score for each patient by applying the following formula:

$$Risk\;score\;=\;\sum_{i=1}^n\beta i\ast i$$

$\beta i$ refers to the coefficients of each gene; $i$ represents the expression value of the gene; and $n$ is the number of genes selected.

### Clinical value of the risk assessment model

The samples were divided into high-risk and low-risk groups by the threshold of median score, and the high- and low-risk groups were further analyzed for differential expression with human leukocyte antigens (HLA) and immune checkpoints.

A nomogram was constructed to calculate an individual’s probability of OS by using the package “rms” of R software. In the nomogram, the samples were scored according to the risk assessment model and clinical indicators. The final sum of the scores was expected to be the corresponding 1-, 3-, and 5-year survival probability. The calibration curve was drawn by comparing the predicted probability of the nomogram with the Kaplan–Meier estimate of the observed survival probability.

### Gene set enrichment analysis (GSEA)

To further research the potential mechanism between diverse risk groups (median value), we performed GSEA [26]. GSEA was performed to find enriched terms that were predicted to have a correlation with the Kyoto Encyclopedia of Genes and Genomes (KEGG) pathway in C2 (“c2.cp.kegg.v7.4.symbols”) [27]. *P* < 0.01 and FDR (false discovery rate) q < 0.05 were considered to indicate statistical significance.

### Identification of potential small molecule drugs

The connectivity map (CMAP) database (http://www.broadinstitute.org) was used to predict potential drugs that may reverse or induce the biological states of GC based on the differentially expressed genes (DEGs). The DEGs were submitted to the CMAP database to search for small molecular drugs that could be used for GC treatment. The enrichment scores ranged from –1 to 1. A negative score suggested that the drug could be beneficial for GC treatment.

### Validation of the gene signature expression

The human gastric mucosa epithelial cell line (GES-1) and six GC cell lines (AGS, HGC-27, MKN-45, SGC-7901, MGC-803 and BGC-823) were cultured in Dulbecco’s modified Eagle’s medium (DMEM) or Roswell Park Memorial Institute (RPMI) 1640 medium with 10% fetal bovine serum following the recommended conditions of cell culture. Total RNA was extracted by using TransZol Up, and cDNA was synthesized and mixed with primers (Supplementary Table S1), and placed on the machine following the manufacturer’s protocols. The relative expression of the gene signature mRNA was analyzed by the 2^−ΔΔCt^ method with glyceraldehyde 3-phosphate dehydrogenase (GAPDH) as the internal reference gene.

Twenty pairs of GC tissues and matched adjacent normal tissues were proceeded to validate the expression of the gene signature mRNA in the same operational and statistical manner as described above.

The protein expression levels of the gene signature were compared between normal and malignant tissues with The Human Protein Atlas (HPA: https://www.proteinatlas.org/).

### Statistical analysis

All statistical analyses were performed by using R 4.0.2. The “limma” package was used to analyze the DEGs between tumor and normal samples. The “Survival” package was used to assess the association of each gene with survival. The Survival predictive accuracy of the risk assessment model was assessed based on a time-dependent ROC curve analysis, and survival rates were calculated using the Kaplan–Meier method. The significance of differences between survival curves was determined using the log-rank test. Student's t-test was used to determine the statistical significance of the differences. *P* values were two-tailed.

## Results

### Identification of the feature genes for modeling

**Fig. 1:**
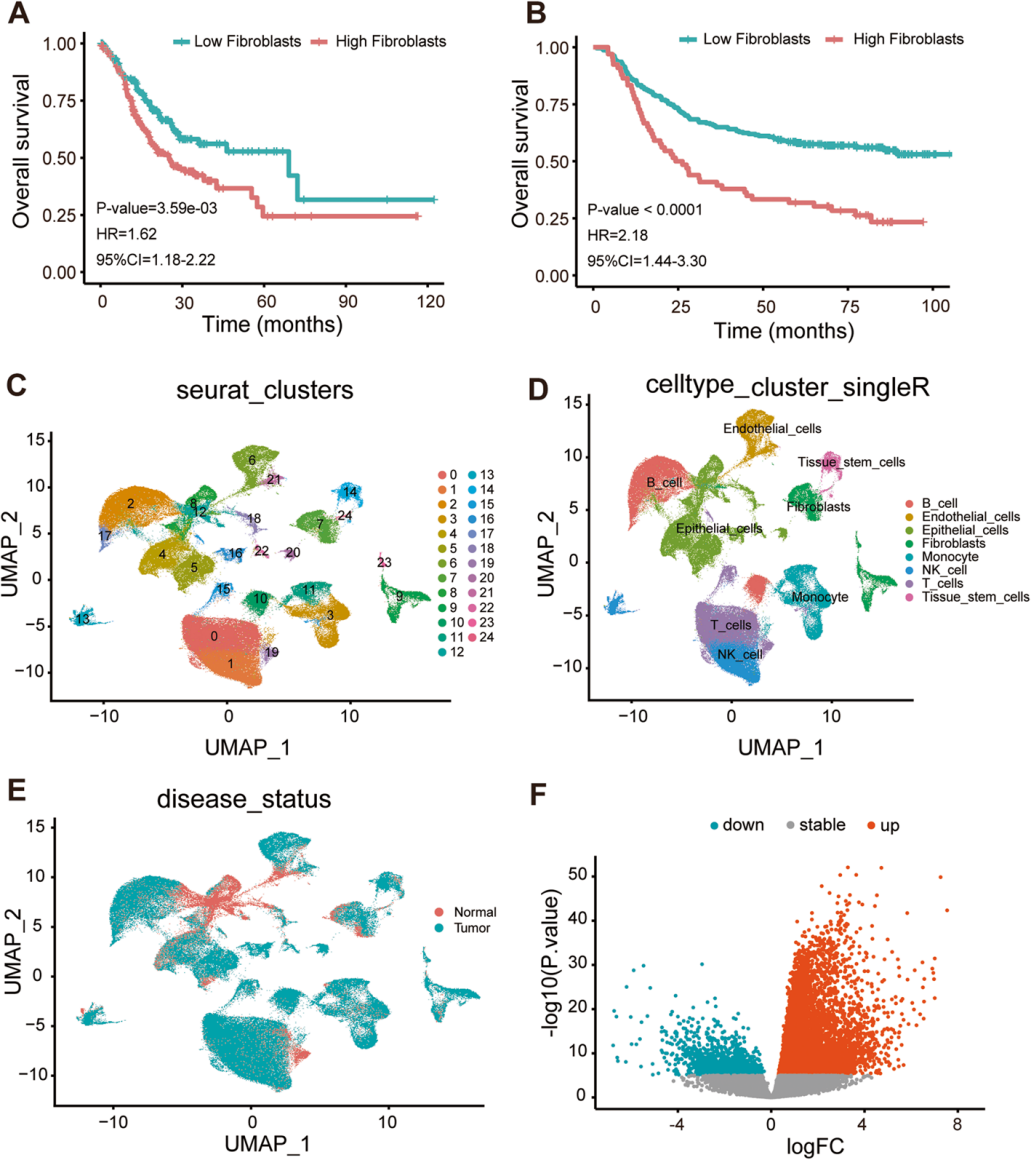
Identification of the feature genes for modeling **A**, **B** The relationship between the abundance of fibroblasts and OS in the TCGA and GSE62254 dataset. **C** Dimensionality reduction and cluster analysis. All cells were clustered into 25 clusters. **D** Annotate cell types according to known cell markers. All cells were annotated into 8 cell types. **E** The status of disease in each cell. All cells were divided into normal and tumor cells. **F** The volcano plot of DEGs in the TCGA dataset

To explore the TME in GC patients, we conducted nontumor cell infiltration analysis using the MCP-counter package and examined the connection between cell abundance and OS in TCGA and GSE62254 datasets. We noticed that the higher the abundance of fibroblasts was, the poorer the survival of patients (Fig. 1A-B). Subsequently, we performed scRNA-seq profiling to identify CAF-related marker genes. The top 10 genes that were significantly differentially expressed in the cell samples were discovered using variance analysis (Figure S1). The genes that were strongly related in each component were screened using the principal component analysis (PCA) approach. Figures S2-3 show a heatmap and dot plot of the top 30 significantly correlated gene.

The first 35 PCs represented the main deviations of the cells (Figure. S4). According to the “UMAP” algorithm and “SingleR”, 158,641 cells were aggregated into 25 clusters and 8 cell types, respectively (Fig. 1C-E). As the cutoff criteria was *P* value = 1.0E-5, a total of 10,640 DEGs between GC tissue of 9822 fibroblasts and normal tissue of 3138 fibroblasts were identified.

In the TCGA dataset, 10,091 DEGs between GC samples and normal samples were obtained by differential analysis, including 1093 downregulated genes and 8998 upregulated genes (Fig. 1F). We regarded the DEGs that had a consistent downregulated/upregulated trend with the single-cell results as the stable fibroblast-associated DEGs. Additionally, by survival analysis, 6389 prognosis-related genes were obtained. From the intersection of the stable CAF-related DEGs and the prognosis-related genes, a total of 280 feature genes overlapped for further modeling (supplementary Table S2).

### A 9-gene risk assessment model for predicting OS

**Fig. 2:**
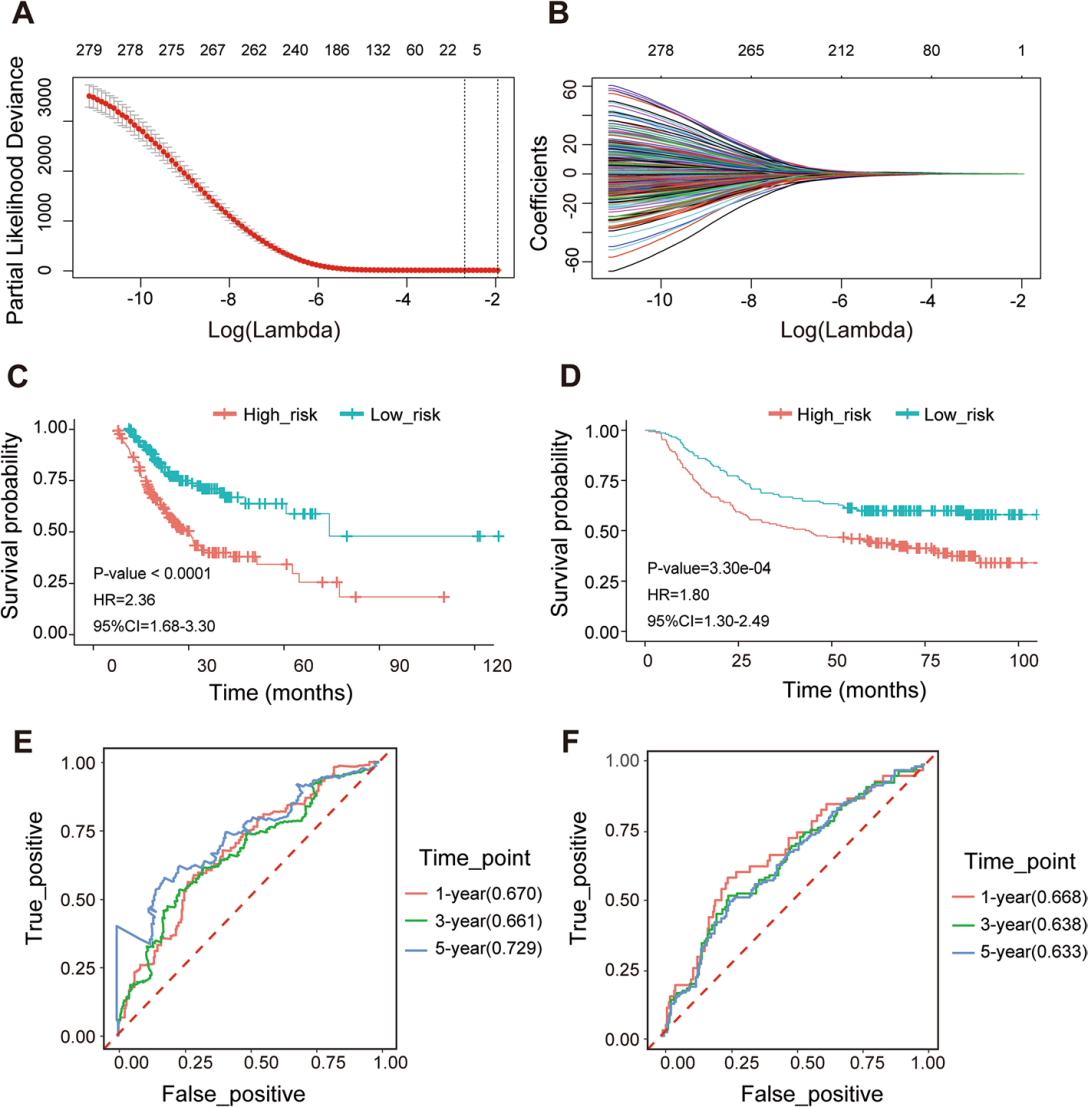
Construction and validation of the prognostic signature. **A**, **B** A 9-gene risk assessment model was constructed by the LASSO Cox regression model. **C**, **D** The Kaplan–Meier survival plots of high-risk and low-risk groups in the TCGA and GSE62254 dataset. **E**, **F** The ROC curves of the prognostic signature in 1-, 3-, and 5-year survival in the TCGA and GSE62254 dataset

We obtained a gene signature containing 9 genes by reducing the dimensionality of these 280 feature genes with the LASSO Cox regression model (Fig. 2A-B). Then, Cox analysis was performed on the 9 genes to construct a risk assessment model. The coefficient of each gene was obtained (supplementary Table S3). Survival analysis based on the median risk score showed that survival time was significantly shorter in the high-risk group than in the low-risk group (Fig. 2C). Simultaneously, we studied the differences in the abundance of the two groups. We found that patients in the high-risk group also had a higher abundance of fibroblasts, which was consistent with our previous results. Then, ROC curves were drawn to verify the risk assessment model, and the AUC values of 1-, 3- and 5-year survival were 0.670, 0.661 and 0.729, respectively (Fig. 2E). In addition, we further validated the model with the GSE62254 dataset (Fig. 2D, F).

### Risk score as a good differentiator and an independent prognostic factor

**Fig. 3:**
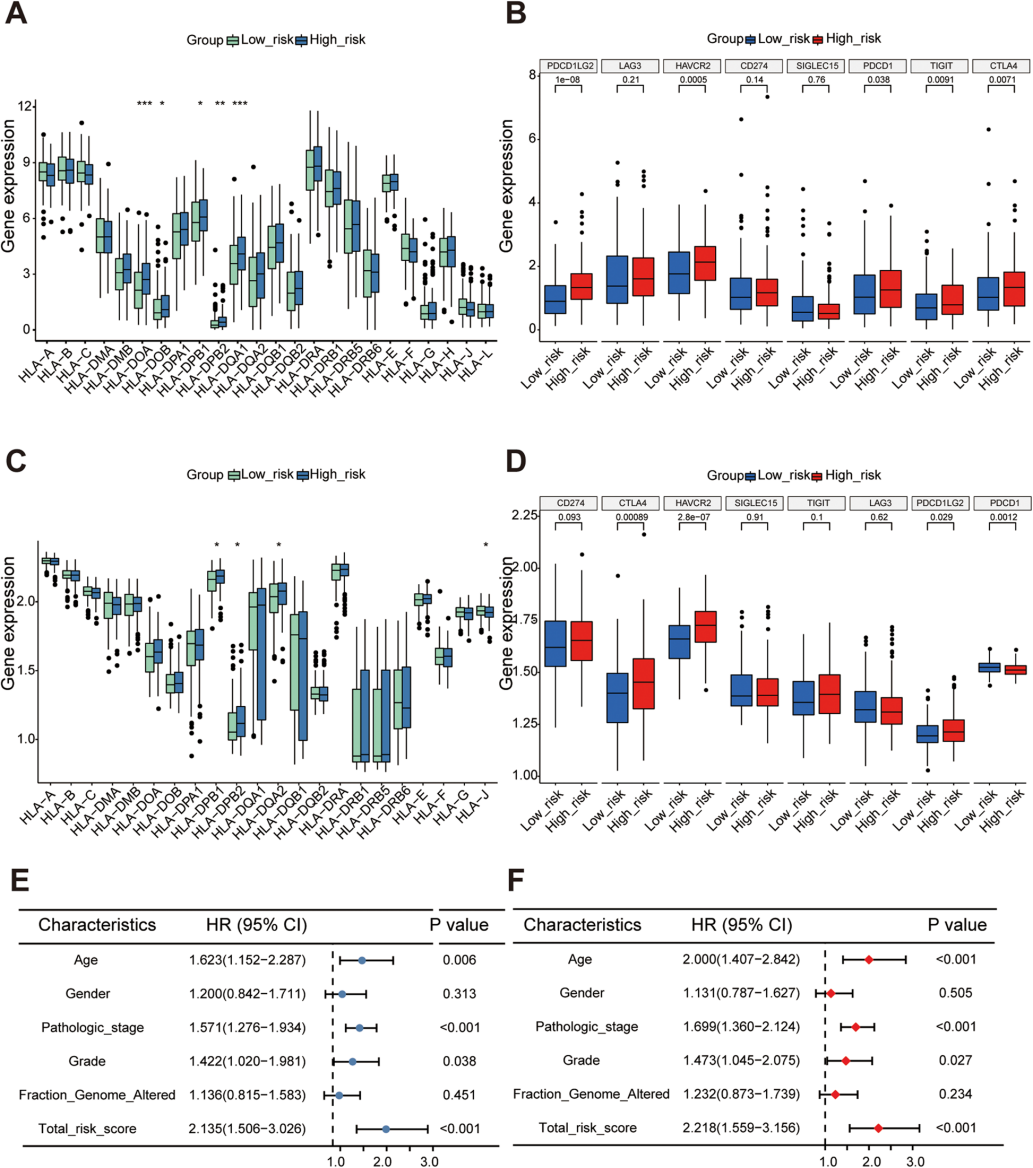
The clinical value of this signature. The boxplot of differences in human leukocyte antigens and high- and low-risk groups in the TCGA and GSE62254 dataset **A**, **C**. The boxplot of differences in checkpoints and high- and low-risk groups in the TCGA and GSE62254 dataset **B**, **D**. Forest plot of prognostic signature and clinical risk factors, the univariate Cox regression analysis in the TCGA dataset **E** and the multivariate Cox regression analysis **F**

The differential expression of human leukocyte antigens (HLA) and immune checkpoints in the high- and low-risk groups was analyzed in the TCGA dataset (Fig. 3A-B) and the GSE62254 dataset (Fig. 3C-D). we could find significant differences in the relevant targets between the high- and low-risk groups.

Univariate and multivariate Cox regression analyses were performed in the TCGA dataset, and the risk score was significantly associated with OS in univariate Cox regression analysis (HR = 2.135, 95% CI = 1.506–3.026, *P* < 0.001; Fig. 3E). Likewise, multivariate analysis showed that the risk score was an independent prognostic indicator in GC (HR = 2.218, 95% CI = 1.559–3.156, *P* < 0.001; Fig. 3F).

### Construction of the nomogram and calibration curves

**Fig. 4:**
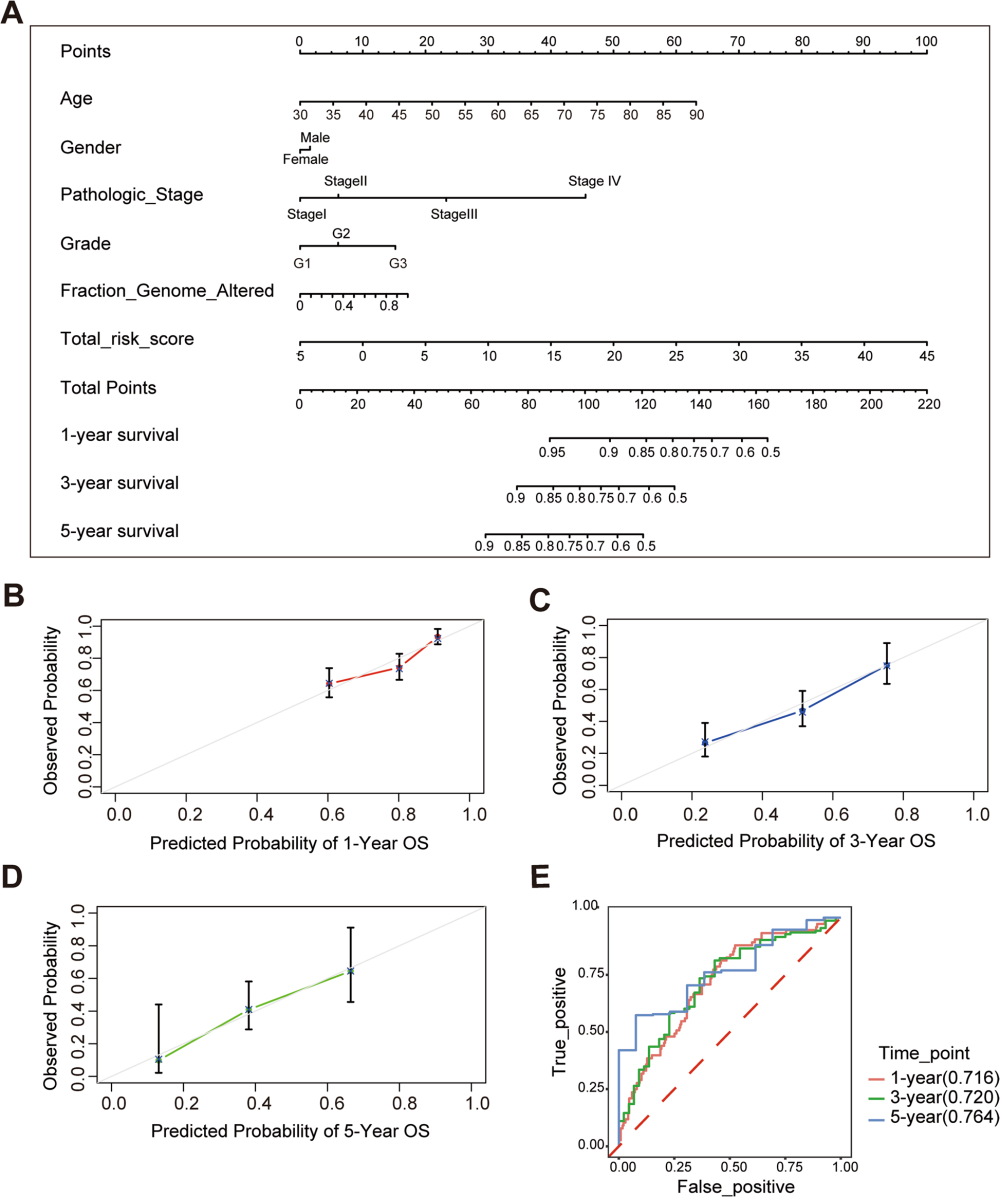
The construction of nomogram. Nomograms for predicting the OS in the TCGA dataset **A**. Calibration curves of nomograms for predicting the OS of 1-, 3-, and 5-year **B**-**D**. The ROC curves of the prognostic signature combined with clinical information in 1-, 3-, and 5-year survival in the TCGA dataset **E**

We constructed the nomogram by combining the risk score with other clinicopathological risk factors. The nomogram showed that our risk score was the most important factor among the various clinical parameters (Fig. 4A). In addition, calibration curves revealed that the predicted and actual survival rates were well matched at 1-, 3-, and 5-years (Fig. 4B-E).

### The risk assessment model had a favorable prognostic prediction in patients with different clinical characteristics

**Fig. 5:**
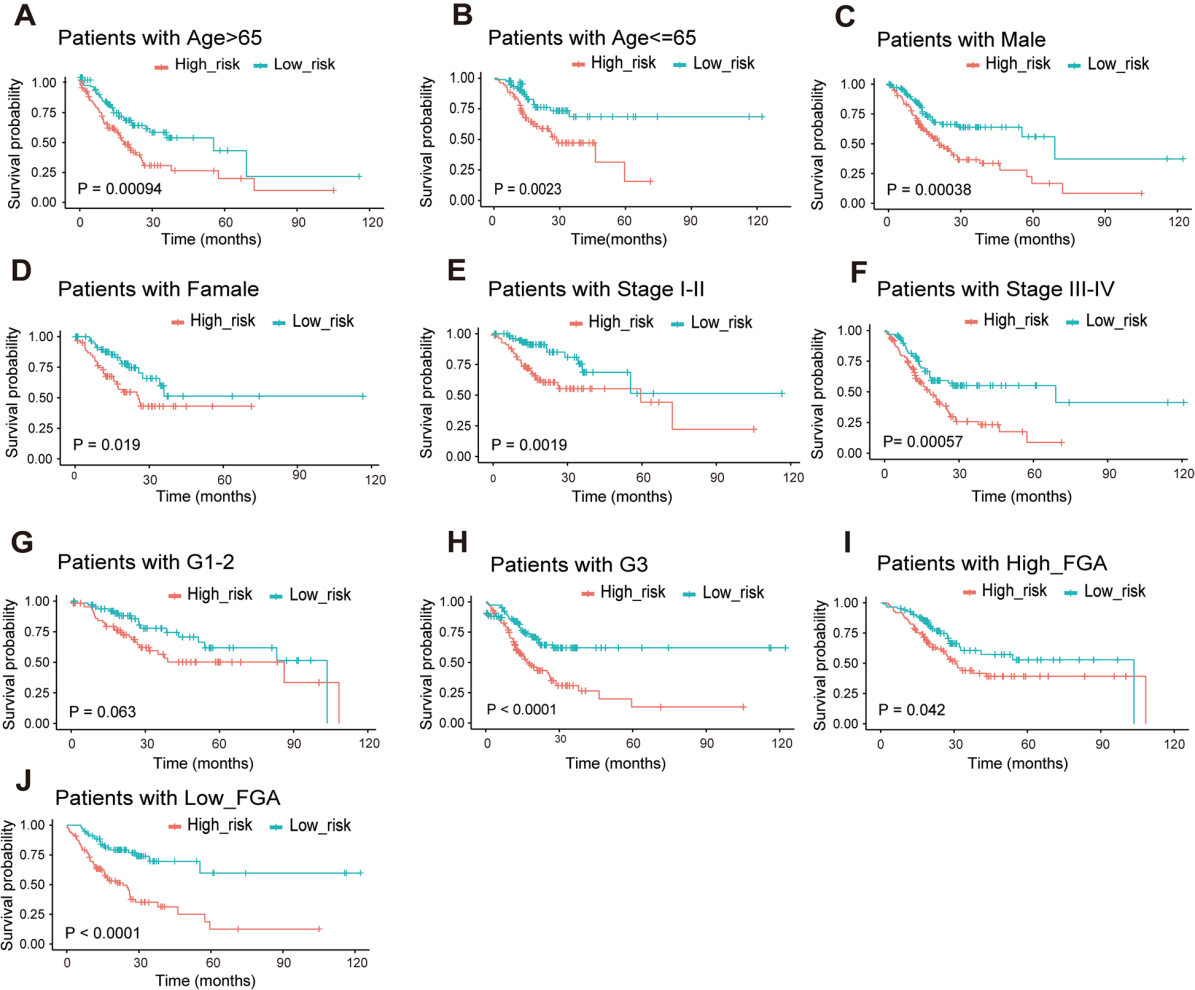
Independent prognostic analysis of the risk assessment model and different clinical characteristics. The Kaplan–Meier survival plots of patients with age > 65 and ≤ 65 **A**, **B**; Males and females **C**, **D**; Stage I-II and Stage III-IV **E**, **F**; tumour grading G1-2 and G3-4(G, H); High-fraction genome altered Low-fraction genome altered **I**, **J**

The risk score was used to predict the OS of the patients according to age, gender, pathologic stage, grade and altered fraction genome. We could see that the survival time of the high-risk group was obviously shorter than that of the low-risk group in each group (Fig. 5A-J). Therefore, we could use the risk assessment model to predict the OS of patients in clinical practice, providing a strong reference for doctors to adjust the treatment promptly.

### GSEA and small molecular drug screening

**Fig. 6:**
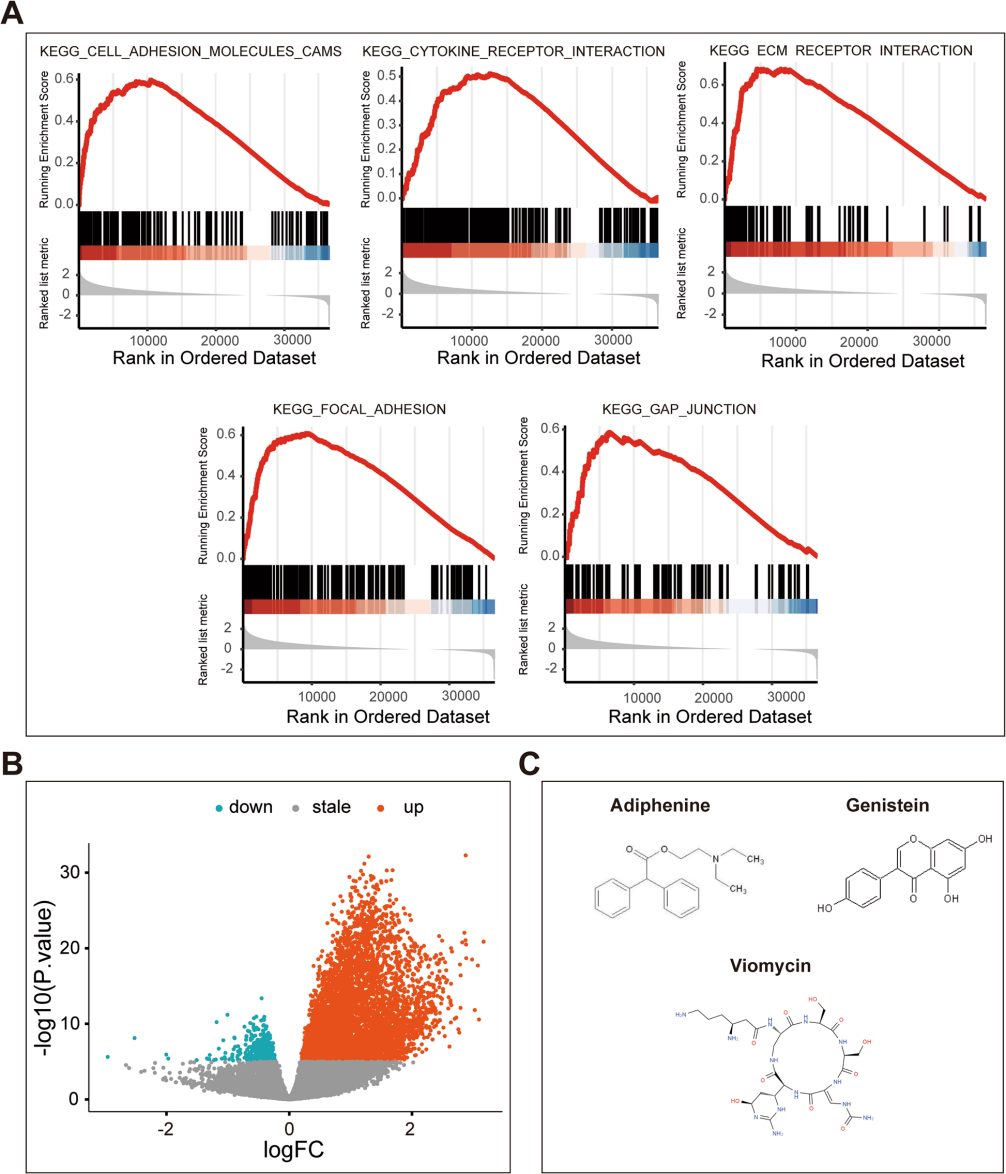
Gene set enrichment analysis and Connectivity map analysis. **A** The upregulated KEGG pathways of top 5 between high-and low-risk groups. **B** The volcano plot of DEGs between high-and low-risk groups. **C** The chemical structures of three small molecule drugs

We performed differential expression analysis between the high- and low-risk groups according to the median risk score. A total of 5803 DEGs, including 439 downregulated DEGs and 5364 upregulated DEGs, were finally identified using the screening criteria: P value < 1E-5 (Fig. 6B). We used the GSEA method to analyze a whole-genome dataset of GC samples between the different risk groups to further understand the molecular mechanism. GSEA analysis using c2 as a reference gene set revealed that biological processes of ECM receptor interaction, focal adhesion, gap junction, cell adhesion molecules cams and cytokine receptor interaction were significantly related to the high-risk group (Fig. 6A).

All the DEGs related to risk score were divided into upregulated and downregulated groups, which were uploaded to the CMAP database. Three small molecular drugs with anticancer properties on GC progression were identified (enrichment score < 0 and *p* < 0.0001): genistein, adiphenine and biomycin (Supplementary Table S4). The chemical structures of these drugs are shown (Fig. 6C).

### Validating the Expression of nine Genes

**Fig. 7:**
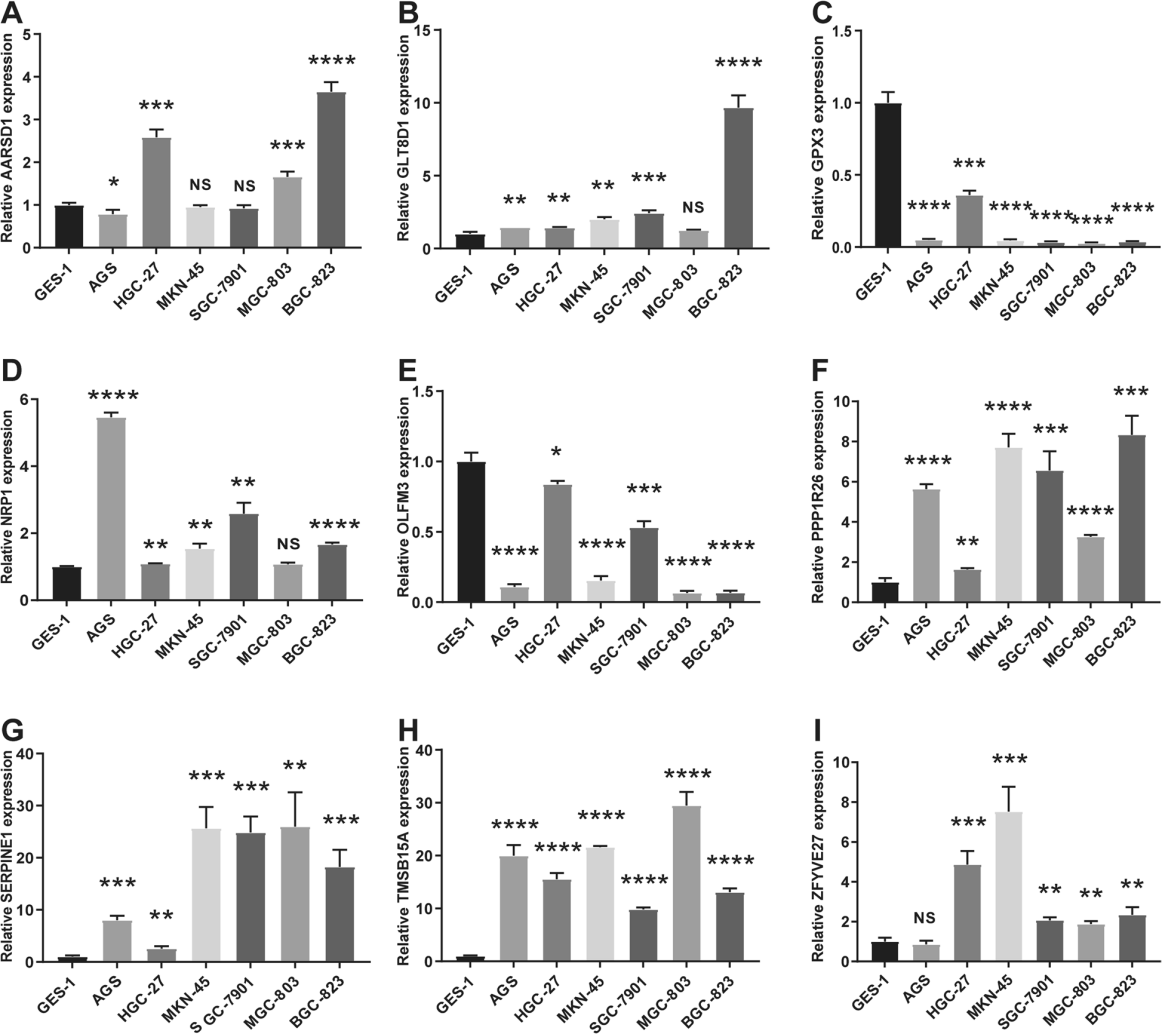
Relative mRNA expression levels of the prognostic genes in GC cell lines and the human gastric mucosa epithelial cell line (GES-1) using qRT–PCR. **A**: for AARSD1; **B**: for GLT8D1; **C**: for GPX3; **D**: for NRP1; **E**: for OLFM3; **F**: for PPP1R26; **G**: for SERPINE1; **H**: TMSB15A; **I**: for ZFYVE27

**Fig. 8:**
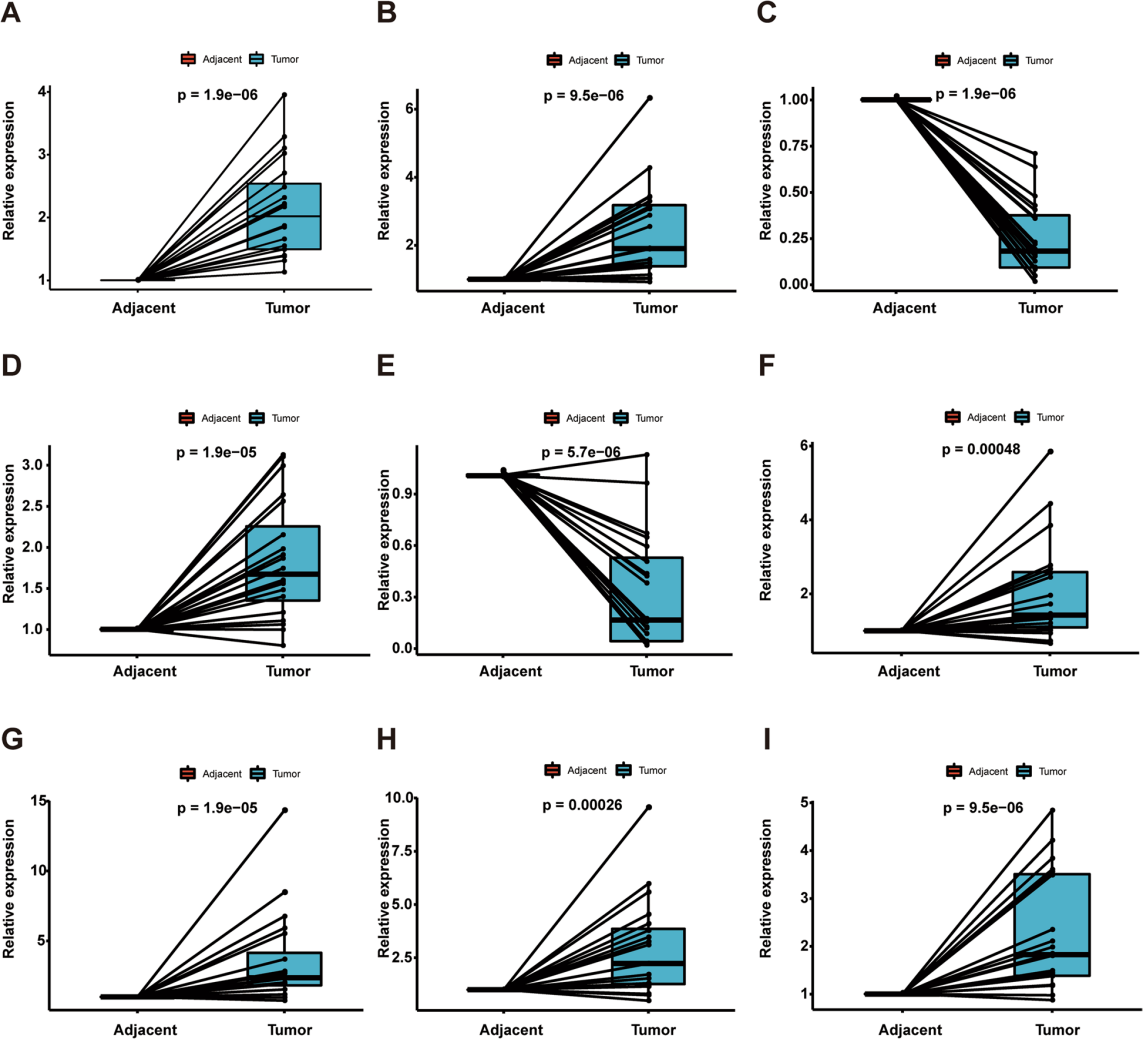
Relative mRNA expression levels of the prognostic genes in GC tissues using qRT–PCR. **A**: for AARSD1; **B**: for GLT8D1; **C**: for GPX3; **D**: for NRP1; **E**: for OLFM3; **F**: for PPP1R26; **G**: for SERPINE1; **H**: TMSB15A; **I**: for ZFYVE27

**Fig. 9:**
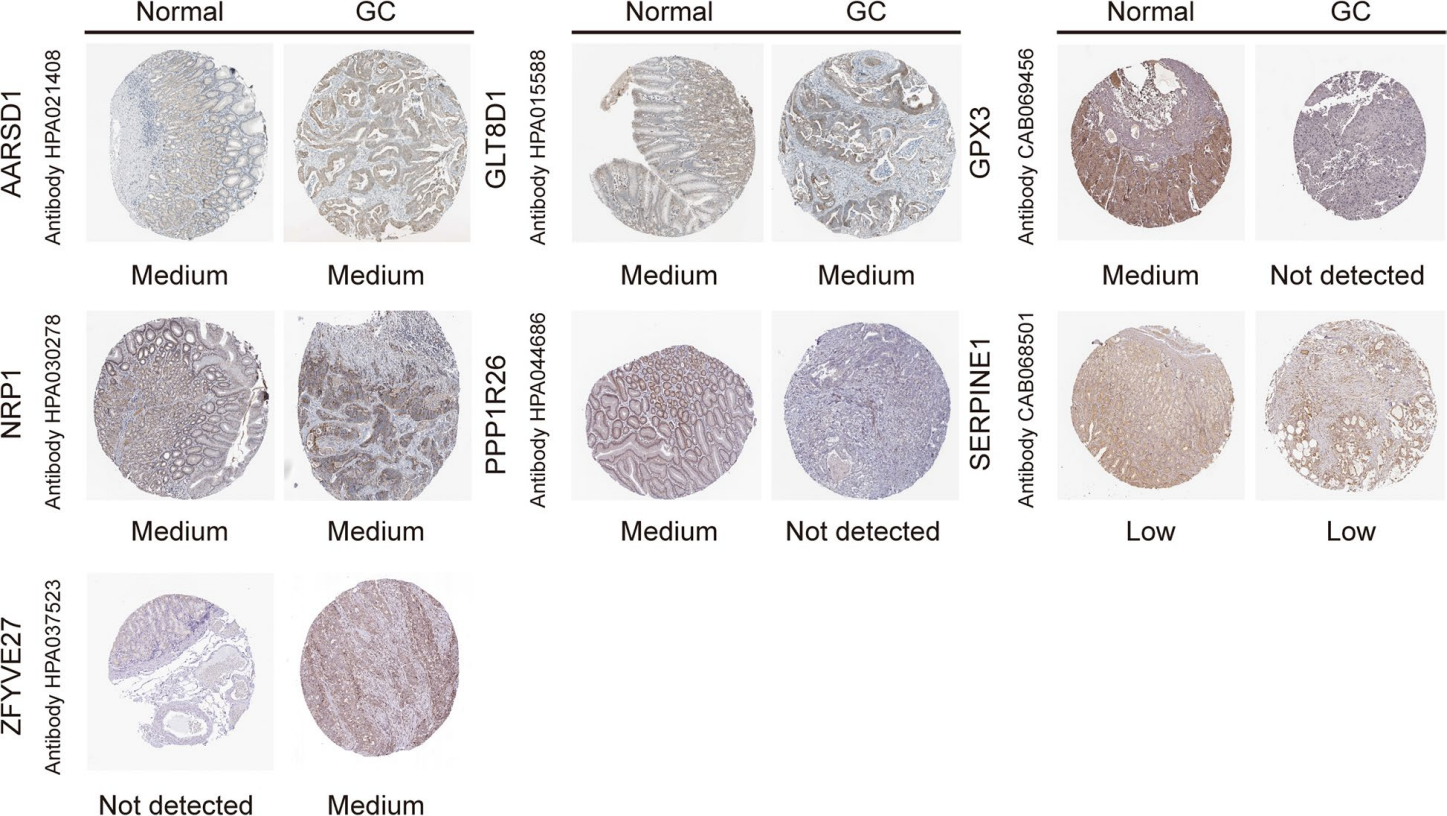
Immunohistochemistry staining of prognostic genes extracted from the HPA database in GC and normal stomach tissue

We used quantitative reverse transcription PCR (qRT–PCR) assays to compare the expression of the gene signature in GC cell lines and normal cell line. Different differential expression can be found in different cell lines, and the results are detailed in Fig. 7. The mRNA levels of GLT8D1, NRP1, PPP1R26, SERPINE1, TMSB15A and ZFYVE27 showed high expression overall, and AARSD1 was highly expressed only in HGC-27, MGC-803 and BGC-823 cells, yet GPX3 and OLFM3 were significantly lower in GC cell lines. Again, as shown in Fig. 8, there is a significant difference in the relative expression levels of these genes in tumor tissue and matched adjacent normal tissues. In addition, immunohistochemical staining was performed to confirm the protein expression of the gene signature using the HPA online site. We compared the differential expression of seven target genes in normal and malignant tissues, except for TMSB15A and OLFM3. The degree of staining for GPX3 and PPP1R26 was stronger in normal tissues than in cancer tissues; on the other hand, ZFYVE27 showed the opposite trend (Fig. 9).

## Discussion

Emerging clinical applications of targeted therapy and immunotherapy underscore the importance of the TME, the complex regulatory network of which poses great challenges to therapeutic efficacy. In light of the dominant sector in the TME and its functional heterogeneity, CAFs have gradually become an intense area of research. On the one hand, it is important for further exploration of tumor mechanisms in CAFs to develop more novel therapeutic targets, but the prediction of prognosis is also a vital part of clinical decisions. However, the specific markers and origin of CAFs remain controversial. In this study, by integrated single-cell and RNA sequencing analysis, a novel signature in GC was developed to identify feature genes of CAFs.

CAFs, as the absolute dominant component of the tumor stroma, secrete various components that participate in constituting and remodeling the ECM. We observed that the higher the abundance of fibroblasts was, the poorer the survival of patients with GC. The reason for this may be that the dense ECM forms a physical barrier that promotes tumor progression and prevents drug penetration [28]. As with the results of our analysis, fibroblast content can be utilized to predict prognosis, which has been validated in numerous tumors [29–33]. In particular, there is a highly aggressive subtype of GC with a very poor prognosis –scirrhous gastric cancer (SGC), which is characterized by rapid infiltration and proliferation of tumor cells with extensive stromal fibrosis [34]. In this fibrotic TME of SGC, researchers explored the biological behavior by constructing SGC cell lines and mouse models [35], gradually depicting the crosstalk between tumor cells and CAFs [34, 36].

CAFs have been demonstrated to promote migration and EMT in GC by activating the JAK2/STAT3 signaling pathway through the secretion of IL-6 [5], as well as activation of the ERK1/2-SP1-ZEB2 pathway via the secretion of IL-33 [37]. Other factors induced by CAFs, such as IL-11, IL-22, IL-17a, FGF9, TGFβ1, lumican, LOXL2, SDR1 and CXCL12, are also involved in the migration and invasion of GC [38–40]. Likewise, CAF-derived galectin-1 and HGF can promote angiogenesis, supporting the progression of GC [41, 42]. Acquired drug resistance severely affects patient treatment prognosis. Numerous studies have shown that CAFs play an important role in mediating drug resistance [43]. CAFs can regulate drug resistance via the secretion of the IL-11-mediated gp120/JAK/STAT3/Bcl2 pathway [7], and activate the PI3K/AKT signaling pathway by generating IL-8, which causes NF-B activation and cisplatin resistance [44]. In addition, Yang et al. found that CAFs can promote chemoresistance by mediating VEGF/NRP2 signaling via CXCL12 secretion [45]. Emerging evidence has demonstrated that CAFs can also affect tumor progression and drug resistance by forming extracellular vesicles (EVs). Studies have shown that CD9-positive exosomes generated from CAFs can be taken up by SGC cells, which promote cancer cell migration and invasion by activating the MMP2 signaling pathway [46]. Similarly, exosomal circ_0088300 derived from CAFs promotes GC malignancy by activating miR-1305/JAK/STAT1 [47], and annexin A6 in CAF-EVs induces drug resistance via activation of β1 integrin-FAK-YAP [48]. Nonetheless, CAF-derived exosomal miRNA-34 and miRNA-139 could inhibit the progression of GC [49, 50]. Collectively, the mystery of the diverse biological functions of CAFs is gradually being unraveled, for which we will also further explore the value of their clinical application.

In this study, we performed scRNA-seq profiling to reveal the fibroblast subset and identify CAF-related marker genes. A total of 280 feature genes were obtained with the intersection of the stable CAF-related DEGs and the prognosis-related genes. By LASSO Cox regression, we successfully constructed and validated a novel 9-gene CAF-related signature to predict the prognosis of GC, and the signature was confirmed as an independent predictor of OS by univariate and multivariate Cox regression analyses. Most of these CAF-related genes are associated with tumorigenesis and cancer progression, including GLT8D1 [51], GPX3 [52], NRP1 [53], PPP1R26 [54], SERPINE1 [55], and TMSB15A [56]. Of these, we focused on SERPINE1, one of the genes upregulated in this gene signature, which encodes plasminogen activator inhibitor-1 (PAI-1). Studies confirm that its overexpression is involved in the progression and unfavorable outcomes in various cancers [55]. Sakamoto et al. proved that PAI-1 from CAFs stimulated esophageal squamous cell carcinoma (ESCC) cell migration and invasion through contact with LRP1 via phosphorylation of Akt and Erk1/2 [57]. Furthermore, CAFs induced M2 polarization in macrophages by secreting CXCL12, which in turn induced PAI-1 secretion and enhanced the malignant behavior of HCC [58]. As a result, the gene signature we constructed can serve as a target reference for CAFs in tumor research. However, the detailed mechanisms in GC warrant further investigation.

Subsequently, we tried to discover promising small molecular drugs for gene therapy of CAF-related gene signatures in GC patients. Traditional Chinese herbal extracts have been demonstrated to be effective in slowing the progression of GC. For example triptonide, a small molecule (MW358) extracted from Tripterygium wilfordii Hook F, efficiently inhibits development and metastasis by blocking the oncogenic Notch1 and NF-B signaling pathways [59]. Wang et al. discovered that several natural products inhibit CAF activity in a series of investigations. By rectifying aberrant microRNA expression, astragaloside IV and treponil restricted the malignancy-promoting capacity of CAFs [60, 61]. In contract, Paeoniflorin suppressed the malignancy of CAFs by decreasing its IL-6 secretion [62]. In this study, we screened three small molecular drugs for the treatment of CAFs. The one with the most significant p value is the one we are interested in, Genistein is a phytoestrogen and a naturally occurring chemical constituent found primarily in legumes. It has anticancer properties, and studies have shown that by targeting distinct biological processes, it can suppress the growth of various cancer cells [63]. In regard to GC research, genistein inhibits tumor cell proliferation by suppressing cancer stem cell-like properties and inducing G2/M arrest [64, 65], as well as improving chemotherapy sensitivity by inhibiting ERK1/2 activity [64]. Nevertheless, the practical application of these potentially therapeutic small molecule compounds requires further exploration and validation.

## Conclusions

We identified a novel CAF-related gene signature for GC by integrating single-cell and bulk RNA sequencing analysis, and these differentially expressed genes might become valuable prognostic and therapeutic targets. We also validated them by multiple approaches and successfully screened genistein, adiphenine and viomycin as potential therapeutic drugs for the treatment of GC patients. However, further studies are still needed to validate the specific mechanisms and effectiveness of these differential genes and therapeutic drugs in GC.

## Supplementary Information

**Additional file 1:** **Table S1.** The primer sequences of the CAF-related genesignature.

**Additional file 2:** **Table S2.** The result of feature genes by the intersection ofthe stable CAF-related DEGs and the prognosis-related genes.

**Additional file 3:** **Table S3.** The coefficient of the CAF-related gene signature.

**Additional file 4:** **Table S4.** The predicted result of the CMAP database.

**Additional file 5:** **Figure S1.** The variance analysis for differentiallyexpressed genes across the cell samples. **Figure S2.** The heatmap of top 30significantly correlated gene. **Figure S3.** The dot plot of top 30 significantlycorrelated gene. **Figure S4.** The main deviations of the cells in the first 35 PCs.

## Data Availability

All data generated or analyzed during this study are included in this published article. We downloaded the corresponding public data resources from the UCSC Cancer Genomics Browser (https://xenabrowser.net/datapages/) and the Gene Expression Omnibus (http://www.ncbi.nlm.nih.gov/geo/), including GSE62254 and GSE183904.

## Abbreviations

CAFs: cancer-associated fibroblasts
GC: gastric cancer
TME: tumor microenvironment
ECM: extracellular matrix
scRNA-seq: single-cell RNA sequencing
RNA-seq: transcriptome RNA sequencing
TCGA: The Cancer Genome Atlas
GEO: Gene Expression Omnibus
OS: overall survival
PCA: principal component analysis
LASSO: least absolute shrinkage selection operator
DEGs: differentially expressed genes
HLA: human leukocyte antigen
