# Supplementary material for: Integrated single-cell and bulk RNA sequencing analysis identifies a cancer-associated fibroblast-related gene signature for predicting survival and therapy in gastric cancer
Source: BMC Cancer. 2023 Jan 31;23:108. doi: 10.1186/s12885-022-10332-w (PMC9887891; doi:10.1186/s12885-022-10332-w)
Supplement: Supplementary file 5 — Additional file 5: Figure S1. The variance analysis for differentiallyexpressed genes across the cell samples. Figure S2. The heatmap of top 30significantly correlated gene. Figure S3. The dot plot of top 30 significantlycorrelated gene. Figure S4. The main deviations of the cells in the first 35 PCs. [file 12885_2022_10332_MOESM5_ESM.docx]

**Supplementary information**

Additional files 1: Figure S1.

The variance analysis for differentially expressed genes across the cell samples.


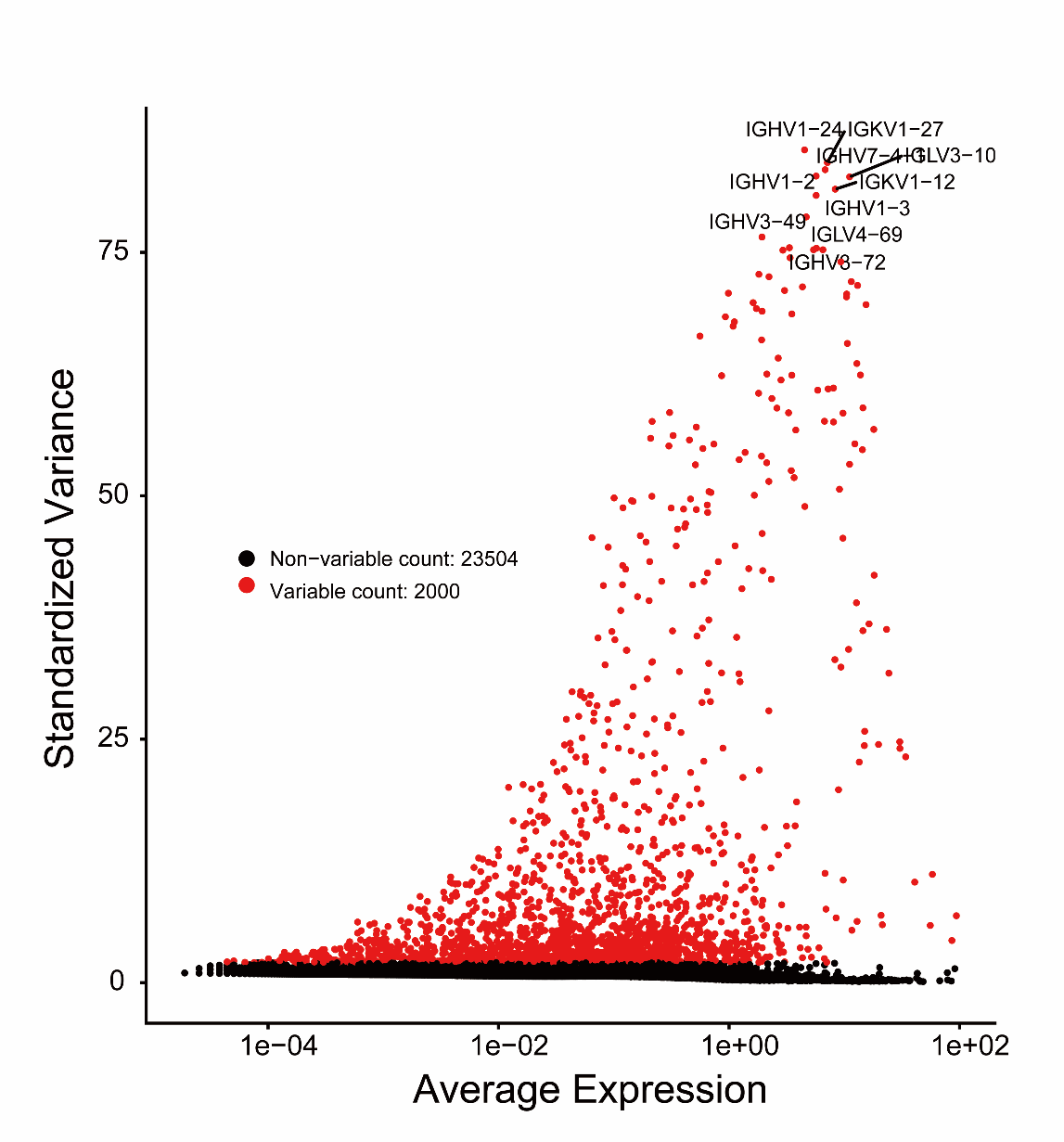


Additional files 2: Figure S2.

The heatmap of top 30 significantly correlated gene.


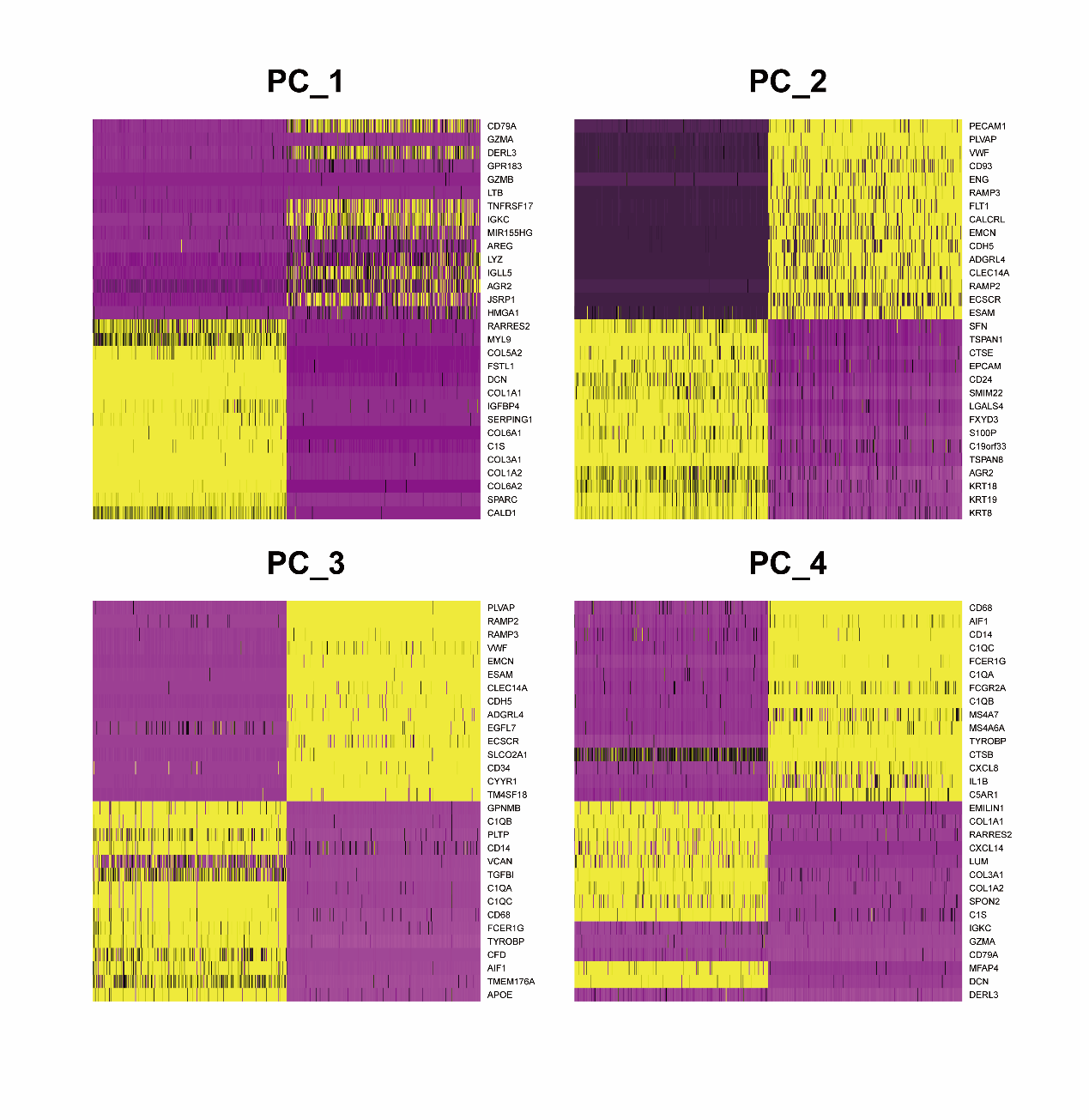


Additional files 3: Figure S3.

The dot plot of top 30 significantly correlated gene.


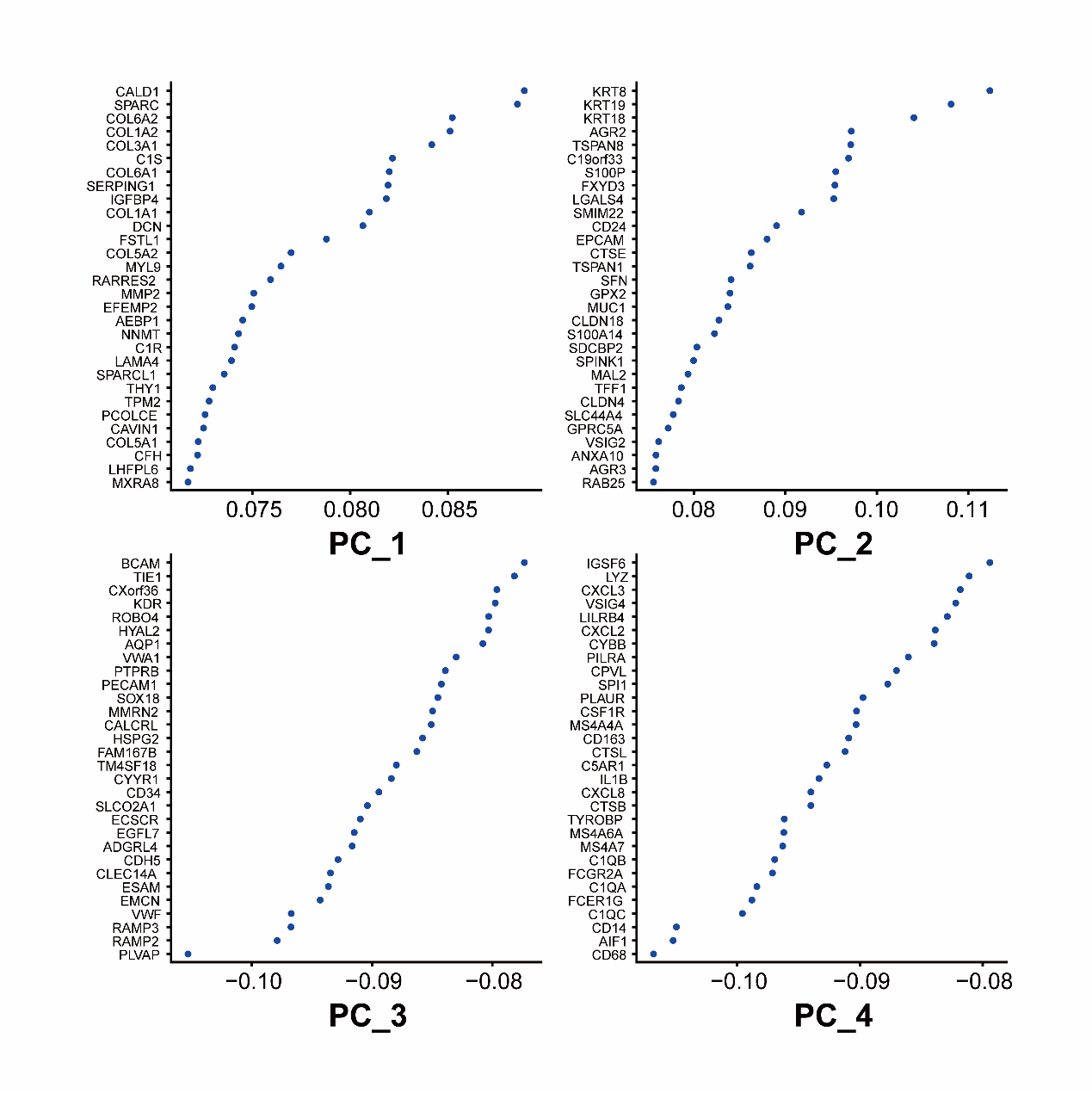


Additional files 4: Figure S4.

The main deviations of the cells in the first 35 PCs.


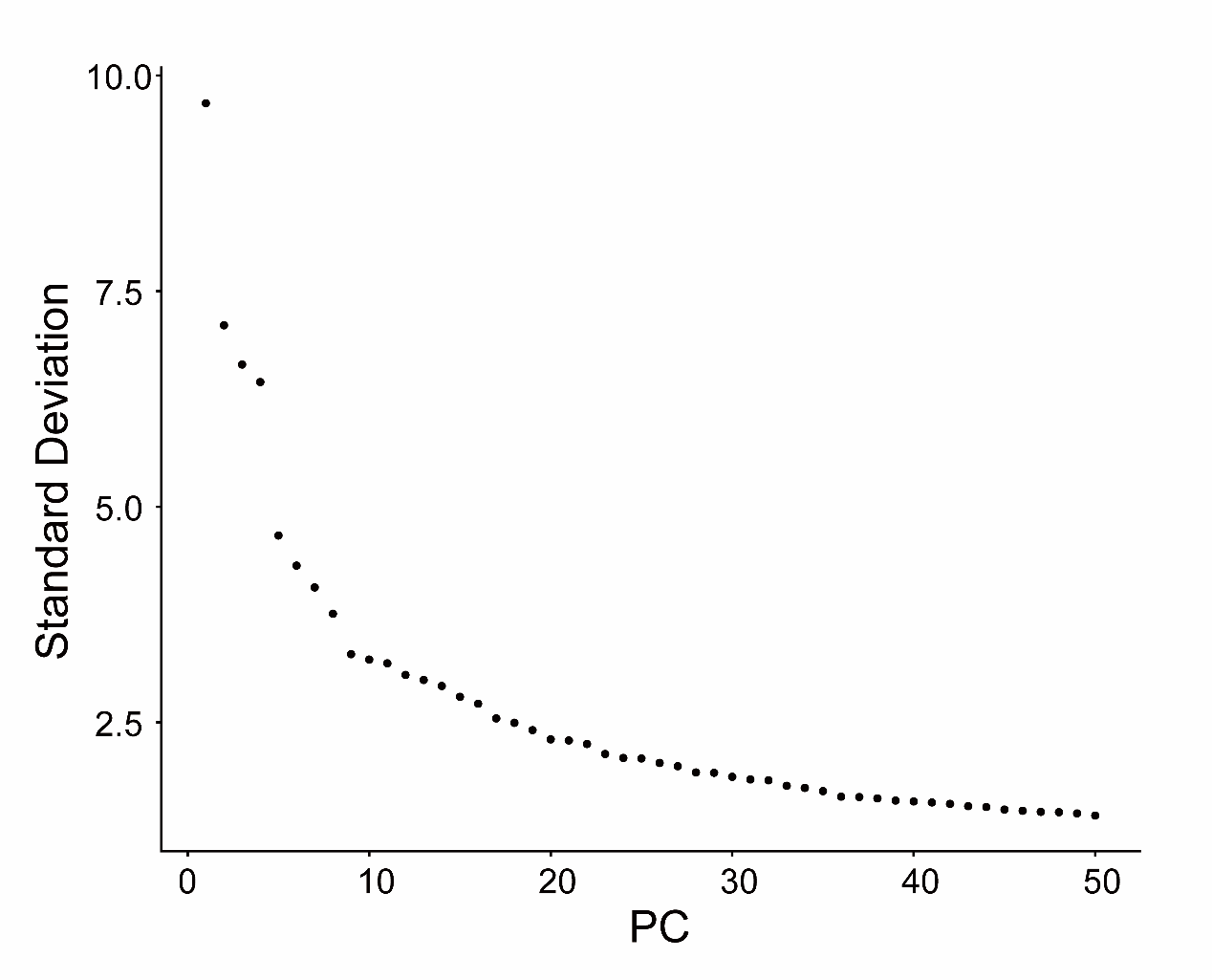


Additional files 5: Table S1.

The primer sequences of the CAF-related gene signature.

Additional files 6: Table S2.

The result of feature genes by the intersection of the stable CAF-related DEGs and the prognosis-related genes.

Additional files 7: Table S3.

The coefficient of the CAF-related gene signature.

Additional files 8: Table S4.

The predicted result of the CMAP database
